# Supplementary material for: Are “Obstetrically Underserved Areas” really underserved? Role of a government support program in the context of changing landscape of maternal service utilization in South Korea: A sequential mixed method approach
Source: PLoS One. 2020 May 6;15(5):e0232760. doi: 10.1371/journal.pone.0232760 (PMC7202644; doi:10.1371/journal.pone.0232760)
Supplement: S1 File — (DOCX) [file pone.0232760.s001.docx]

<Original questionnaires in Korean >

| **보건소 담당자**  **① 관할지역에 대한 질문**   - 귀하의 관할 지역이 분만 취약지 대상이 된 이유가 무엇이라고 생각하십니까? - 분만 취약지 사업의 지원을 받은 지 얼만큼 되셨습니까?   **② 관내 분만기관에 대한 질문**   - 현재 관내 분만 기관이 있는지? - 어떤 유형의 기관입니까? 예시) 대학병원/종합병원/개인 산부인과 병원 혹은 의원/ - 조산원/의료원/기타 - 귀하의 생각에 관내 의료기관에 대한 산모들의 만족도는 어느 정도 된다고 생각하십니까? - 만족한다고 생각한다면 어떠한 요인이 만족하게 만든다고 생각하십니까? - 그렇지 않다고 생각한다면 어떠한 요인이 불만족의 원인이라고 생각하십니까? - 귀하 본인의 평가에 따르면 관내 의료기관의 의료진, 진료 서비스, 시설 및 환경의 수준은 어떻다고 생각하십니까?   **③ 분만취약지 지원사업에 대한 질문**   - 귀하의 관할 지역에서 분만 취약지 지원사업 시행 여부를 검토 시 어떠한 요인들이 고려되었습니까? - 현재 귀하 지역의 분만취약지 지원사업을 통한 지원 병원에 추가적으로 지원이 이루어진다면 가장 우선적으로 지원이 되어야 할 부분이 무엇이라고 생각하십니까? 예시) 우수한 의료진 유치지원/의료분쟁에 대한 보장/첨단 의료기기지원/병원시설개선지원/추가재정지원/기타 - 현재 분만 취약지 지원사업에서 지속되어야 할 부분과 수정되어야 할 부분은 무엇이라고 생각하십니까? - (위의 질문에 대한 답에서 사업의 지속성, 안정성에 대한 답이 나오지 않았을 경우 다시 별도 질문) 분만취약지 사업의 지원을 받아 개선이 되었을 경우, 지원 대상에서 다시 제외되었을 때 오는 불이익에 대해서 우려가 있는지요? - 지속적이고 안정적으로 산부인과들이 개원을 하고 운영을 할 수 있기 위해서는 지원 사업이 어떻게 운영되어야 한다고 생각하는지요? - 귀하가 생각하시는 효과적인 분만 취약지 해결 방안이 있다면 무엇인지요?   **④ 보건소의 모자보건사업에 대한 질문**   - 귀하의 보건소를 포함한 관내 보건소에서 하는 모자보건 사업들은 무엇이 있습니까? 예시) 찾아가는 산부인과/임산부 등록사업/임산부 이송지원 사업/고위험 임산부 등록 사업/산전 검사 및 진료/초음파 검사/산후 우울증 교육 및 스크리닝/철분제, 엽산제 제공/주기적 전화상담/기타 - 이 중 시행이 잘 이루어지고 있는 사업들은 무엇이며 그 요인이 무엇이라고 생각합니까? - 이중 잘 시행이 안 되고 있는 사업들은 무엇이며 그 이유가 무엇입니까? - 현재 관내 보건소에서 시행하고 있는 출장 장려정책은 무엇이 있습니까? - 예시) 산모 및 신생아 도우미사업/초음파할인 및 무료쿠폰 제공 /모자보건 수첩발급/청소년 산모 임산출산의료비 지원사업/출산장려금 지원사업/신생아 청각선별검사사업/없음/기타 - 귀하의 지역에서 관내에 분만 병원이 없거나 적은 이유는 무엇이라고 생각하십니까? (혹은 관내 분만 병원이 있지만 관내 분만건수가 매우 낮은 이유가 무엇이라고 생각하십니까?) - 관내에 산모 전용 이송 수단이 있습니까?   ※ 가장 이상적인 분만 환경은 어떠한 것이라고 생각하십니까? |
| --- |

| **대상 지역 산모 (지원 병원 이용/미이용)**  **① 지원사업 이후 산전 진찰/출산 경험에 관한 질문**  ∙ 몇 번째 출산이었습니까?  <산전 진찰>  ‐ 어디서 산전 진찰하셨습니까?  ‐ 그 곳을 선택하신 이유는?  ‐ 귀하의 집에서 귀하가 받으신 산전 진찰 기관까지 소요되는 시간은?  ‐ 귀하의 집에서 가장 가까운 산전 진찰 기관까지 소요되는 시간은?  ‐ (해당지역이 아닌 인근지역에서 진찰을 받은 경우) 거리가 멀어서 불편하지는 않았는지요? 해당지역의 산부인과와 비교 시 어떤 장단점이 있었는지요?  ‐ 산전 진찰 시 특이사항이 있었습니까? (태아 혹은 산모에서의 이상 감지)  ‐ 그러한 상황에 대해서 어떻게 하셨는지요?  <출산>  ‐ 어디서 출산을 하셨는지요?  ‐ 그 곳을 선택하신 이유는? (산전 진찰 기관과 동일기관인 경우/다른 기관인 경우)  ‐ 귀하의 집에서 귀하가 출산한 기관까지 소요되는 시간은?  ‐ 귀하의 집에서 가장 가까운 분만 기관까지 소요되는 시간은?  ‐ (해당지역이 아닌 인근지역에서 분만을 한) 거리가 멀어서 불편하시지는 않았는지요? 해당지역의 산부인과와 비교 시 어떤 장단점이 있었는지요?  ‐ 출산 시 특이사항이 있었는지요? 예시) 유산 가능성, 고령임신, 임신중독증, 임신성 당뇨, 조기진통 가능성 등  ‐ 그러한 상황에 대하여 어떻게 하셨는지요?  **② 분만취약지 지원사업에 대한 질문**   - 귀하는 귀하의 거주지 지역이 분만 취약지 지원사업으로 지정되어 지원을 받는 사실을 알고 계십니까? - 알고 계시다면 어떠한 사업들이 시행되는지 인지하고 계신 것들을 말씀해주세요 - 분만 취약지 지원사업을 통해 조성된 관내 산부인과를 이용해보신 경험이 있으십니까?   ‐ Yes ⇨ 이용해 보셨다면 만족도는 어떠셨나요?  ‐ No ⇨ 이용하지 않으신 이유가 무엇인가요? (예시: 의료진에 대한 신뢰도, 병원의친절도, 위급상황에 대한 대처 능력, 병원의 의료시설 및 환경, 산후조리 환경 등)  (현재 분만 취약지 지원사업을 통해 이루어지고 있는 사업들을 소개해 준 후)   - 다른 분만 취약지 지원사업들 중 시행이 잘 이루어지고 있는 사업들은 그 성공요인이 무엇이라고 생각합니까? - 다른 분만 취약지 지원사업들 중 잘 시행이 안 되고 있는 사업들은 그 이유가 무엇입니까? - 현재 분만 취약지 지원사업에서 지속되어야 할 부분과 수정되어야 할 부분은 무엇이라고 생각하십니까?   ※ 가장 이상적인 분만 환경은 어떠한 것이라고 생각하십니까? |
| --- |

| **지원 대상병원 산부인과 의사, 병원 담당자**  **① 분만취약지 지원사업에 관한 질문**   - 현 귀하의 근무지역이 분만 취약지로 선정되어 분만취약지 지원사업이 시행되고 있는 것을 알고 계시는지요? - (현재 분만 취약지 지원사업을 통해 이루어지고 있는 사업들 소개해 준 후) - 다른 분만 취약지 지원사업들 중 시행이 잘 이루어지고 있는 사업들은 그 성공요인이 무엇이라고 생각합니까? - 다른 분만 취약지 지원사업들 중 잘 시행이 안 되고 있는 사업들은 그 이유가 무엇입니까? - 현재 분만 취약지 지원사업에서 지속되어야 할 부분과 수정되어야 할 부분은 무엇이라고 생각하십니까? - (위의 질문에 대한 답에서 사업의 지속성, 안정성에 대한 답이 나오지 않았을 경우 다시 별도 질문) 분만취약지 사업의 지원을 받아 개선이 되었을 경우, 지원 대상에서 다시 제외되었을 때 오는 불이익에 대해서 우려가 있는지요? - 지속적이고 안정적으로 산부인과들이 개원을 하고 운영을 할 수 있기 위해서는 지원 사업이 어떻게 운영되어야 한다고 생각하는지요?   **② 개원병원 운영환경에 관한 질문**   - 개원 병원 소재지 주소(봉직의 경우 근무 병원 소재지 주소) - 개원한지 얼마나 오래되었습니까? (___년) (봉직의 경우 근무 년 수) - 개원(봉직)의 경력 기간/해당 기관 개원(봉직) 기간 - 병원 전체의 의료진 수는 몇 명입니까 (과별 의사 수, 간호사수, 기타 직원 수) - 월평균 외래 방문 산모 수는? - 월평균 출산 건수는? - 귀하는 귀하의 거주지 지역에서 병원을 운영(혹은 근무)하면서 가장 큰 어려운 점이 무엇인지요? - 귀하의 병원에서 최근 5년 이내 의료사고/소송 건수는 몇 차례 있었습니까? - 있었다면, 어떤 종류의 사고였는지요? (예시: 산모 혹은 신생아의 사망, 산모 혹은 신생아의 합병증 등) - 사고의 원인은 무엇이었는지요? (예시: 불가항력적 사고, 의료진 부족, 시설 및 자원 부족, 신속한 환자 이송 시스템 부족, 기타) - 그 일이 병원의 운영에 어떠한 영향을 미쳤습니까? (ex: 분만 건수 감소, 보상 및 소송으로 인한 경제적 손실, 자신감 및 의욕 상실 등등) - 지난 3년 동안 귀하의 병원에서 산모를 상급병원으로 전원 해야 하는 경우는 어떠한 경우였고, 몇 회 정도 있었습니까? - 지난 3년 동안 귀하의 병원에서 신생아를 상급병원으로 전원해야 하는 경우는 어떠한 경우였고, 몇 회 정도 있었습니까? - 다른 어려움들이 보상될 수 있는 수준의 합리적인 월수입은 얼마라고 생각하십니까? ____ 만원 - 장기적인 병원 운영을 계획할 수 있는 수준의 합리적인 당직횟수는 월 몇 회라고 생각하십니까? ___ 회 - 적정한 의료의 질을 갖춘 분만을 위해서 추가로 추가되어야 할 의료진의 수는 몇 명이라고 생각하십니까? (산부인과 의사/타과의사/간호사/기타 직원 별도로 응답), 그러한 의료진이 왜, 어떤 경우에 추가로 더 필요하다고 느끼셨는지? - 최근 분만 취약지의 산부인과 의료수가가 200% 인상된 바 있습니다∙ 이것이 수익에 어느 정도 영향을 미치는지요? (실수익의 몇 %정도가 증가하였는지요?) 이 인상정책이 현 지역에서 지속적으로 병원을 운영하는데 유인요인으로 작용할 수 있을까요? - 가장 이상적인 분만 환경은 어떠한 것이라고 생각하십니까? |
| --- |

<Questionnaires translated in English>

| **Public health center staffs**   1. Questions for area  - Why do you think your area is subject to OUAs? - How long have you been supported by government OUA support program?  1. Questions for maternal hospital  - Is there a maternal hospital in the area? - What type of institution? ex) University hospital/General hospital/private gynecology hospital or clinic / Midwifery, etc∙ - In your opinion, what is the satisfaction level of mothers with maternal hospital in the area? - If you think they are satisfied (or unsatisfied), what factors do you think make them satisfied (unsatisfied)? - According to your own evaluation, what is the quality of medical staff, medical care services, facilities, and overall environment?  1. Questions for government OUA support program  - What factors are taken into account in decision making of government-support program in your area? - If there is additional support, what do you think should be the first priority? - What do you think should be sustained and revised in the current government support program? - If the improvement has been made with the government support program, is there concern about the possibility of being excluded from the support program? - How do you think the support business should be run in order to allow the maternal hospital to operate steadily and stably?  1. Questions for maternal and child program of health center∙  - What kind of maternal and child health projects are being conducted in public health centers in the area including yours? - Which of these are performing well, and what do you think is the factor? - What projects are not performing well and why? - Why do you think there are no or fewer maternal hospitals in your areas? - Or why do you think the delivery number in your area is still very low even if there is a government support maternal hospital in the area? - What do you think is an ideal delivery environment? |
| --- |

| **Mothers in OUAs**  **(who have utilized government-supported hospitals / who have not)**   1. Questions about prenatal care and childbirth experience since the start of the government support program  - How many times did you have childbirth? - Prenatal consultation - Where did you go for prenatal care? - Why did you choose it? - How long did it take from your home to the prenatal institution? - How long does it take from your home to the nearest prenatal care center? - (if you used the hospital for prenatal care in the neighboring city) Did you feel inconvenient because of the distance? What are the pros and cons compared to the maternal hospital in your area? - Childbirth - Where did you give birth? - Why did you choose that place? - How long did it take from you home to that place? - How long did it take to get to the nearest maternal hospital from your home? - (if you used the hospital for delivery in neighboring city) Did you feel inconvenient because of the distance? What are the pros and cons compared to the maternal hospital in your area? - Was there anything unusual about giving birth? Ex) possibility of miscarriage, advanced pregnancy, toxemia, etc∙ - What did you do about it?  1. Questions about government support program  - Did you know that your area of residence is designated and supported as an OUA? - If yes, please tell us what you know about the support program∙ - Have you ever utilized the maternal hospital supported by the government? - Yes => how was your satisfaction? - No => why did you not use it? - (After giving detailed information on government support program) among the program components, what do you think should be sustained and revised? - What do you think is the ideal delivery environment? |
| --- |

| **Doctors, nurses, and managers**  **in government-supported maternal hospitals**   1. Questions regarding the operating environment of the hospital  - How long has it been since opening? - Number of working years in government-supported maternal hospital - Number of working years in the respondent’s whole career - How many medical staffs are in the whole department of OB/GY? - What is the monthly average number of delivery and outpatient visits? - What is the biggest challenge working in government-supported hospital? - How many medical accidents/ lawsuits have occurred in your hospital within the last 5 years? - If yes, what kind of accidents was it? And what was the cause of it (lack of medical staff, lack of equipment, etc∙)? - How did that affect the hospital’s operation? (ex: economic loss due to litigation or compensation, loss of confidence and motivation, etc∙) - In the past 3 years, what was the case where you have to refer the mother to higher-level hospital, and how many times did that happen? - In the past 3 years, what was the case where you have to refer the newborn to higher-level hospital, and how many times did that happen? - What is your reasonable monthly income level that can compensate for other difficulties? - How many times of duty per month do you think is reasonable enough to plan long term employment? - How many medical staff do you think need to be added for the right quality of delivery (doctors, nurses, or other employees, separately)  1. Questions about government support program  - Among the program components, what do you think should be sustained and revised? - If the improvement has been made with the government support program, is there concern about the possibility of being excluded from the support program? - How do you think the support business should be run in order to allow the maternal hospital to operate steadily and stably? |
| --- |
