# Supplementary material for: Are “Obstetrically Underserved Areas” really underserved? Role of a government support program in the context of changing landscape of maternal service utilization in South Korea: A sequential mixed method approach
Source: PLoS One. 2020 May 6;15(5):e0232760. doi: 10.1371/journal.pone.0232760 (PMC7202644; doi:10.1371/journal.pone.0232760)
Supplement: S1 Table — (DOCX) [file pone.0232760.s002.docx]

Supplemental table 1∙ Characteristics of interviewees

| Interviewees | N | % of female |
| --- | --- | --- |
| Mothers | 42 | 100% |
| Maternal and child health staffs in local health centers | 18 | 88∙9% |
| Hospital administrators* | 17 | 5∙9% |
| Obstetricians* | 18 | 5∙6% |
| Nurses* | 8 | 100% |
| Total | 103 | 66% |

*Working in government-supported hospital
