# Supplementary material for: Are “Obstetrically Underserved Areas” really underserved? Role of a government support program in the context of changing landscape of maternal service utilization in South Korea: A sequential mixed method approach
Source: PLoS One. 2020 May 6;15(5):e0232760. doi: 10.1371/journal.pone.0232760 (PMC7202644; doi:10.1371/journal.pone.0232760)
Supplement: Attachment — Submitted filename: PONE-D-19-31283 comments.docx [file pone.0232760.s003.docx]

**Reviewer’s report:** Richard Kalisa MD, PhD

Thank you for the opportunity to review this manuscript

The manuscript provides an in-depth evaluation of the effectiveness of the government support program for obstetrically underserved areas in South Korea. It highlights the facilitators and barriers to use of government-supported hospital and the importance of the area-level in explaining the maternal health services utilisation in OUAs. The findings are useful to the program and could support its continued implementation and improvement. Overall, the paper is well-written. However, a few issues need consideration:

1. Qualitative arm of the study: The authors should provide details on the sampling of the study participants, rigour/trustworthiness of their qualitative study findings and provide illustrative quotes for supply-side barriers to the success of the program. In addition, under “preference for a specialised maternal hospital over a general hospital” they should label the quote appropriately.
2. In the discussion, it would be important to see a comparison between this study findings and previous evaluations conducted on the program. What are some of the similarities and differences among the different evaluations – findings and recommendations?
3. Results: Revise the sentence “Specifically….” to read better
4. Discussion: The recommendation on “…*just making their travel more comfortable by providing transportation itself or subsidizing transportation expenses rather than persuading them to use local hospital in their residential area would be more reasonable way to ensure accessibility while better serving their needs*.” might negate the importance of physical/geographical accessibility. Also, could women still utilising city hospital be related to birth preparedness and complication readiness?
5. The manuscript needs an entire revision of typographical errors
6. Add the study strength and suggestions for further studies
7. The authors should consider using PLoS ONE reference style
