# Supplementary material for: Are “Obstetrically Underserved Areas” really underserved? Role of a government support program in the context of changing landscape of maternal service utilization in South Korea: A sequential mixed method approach
Source: PLoS One. 2020 May 6;15(5):e0232760. doi: 10.1371/journal.pone.0232760 (PMC7202644; doi:10.1371/journal.pone.0232760)
Supplement: Attachment — Submitted filename: Rebuttal letter_final.docx [file pone.0232760.s004.docx]

Journal Requirements:

 When submitting your revision, we need you to address these additional requirements:

1. Please ensure that your manuscript meets PLOS ONE's style requirements, including those for file naming. The PLOS ONE style templates can be found at <http://www.plosone.org/attachments/PLOSOne_formatting_sample_main_body.pdf> and <http://www.plosone.org/attachments/PLOSOne_formatting_sample_title_authors_affiliations.pdf>

Response) Thanks. We have checked the submission guidelines you attached and revised accordingly.

 2. Please address the following:

- Please ensure you have thoroughly discussed any potential limitations of this study within the Discussion section.

Response) Thanks for your comments. We have added the limitations as below (from line 510 to 526):

“There are several limitations to be considered when interpreting the results. First, we used the information on the national health insurance premium as a proxy for economic status because NHIS data does not have the information on the actual income. Korean National Health Insurance premium is based on two contribution scheme: 1) for industrial workers and government and school employees, the contribution is proportional to wage income 2) for the self-employed, the contribution is calculated with a formula based on income and property because the incomes of the self-employed are only partially captured. Therefore, there might be a mismatch between the insurance premium and actual economic status. However, we assume that error between captured by insurance premium and actual income would be minimal. Also, we operationalized variable as quintile rather than continuous one so chance of miscategorization into the wrong wealth quintile would be low. Another limitation lies in the fact that although interviewing a diverse range of individuals enables triangulation of collected information, and thus allows a better assessment of the generality of the explanation that one develops, we could not confirm that the mother interviewees were from various background such as socioeconomic status because we recruited them indirectly through the government-supported hospitals or public health center staffs. However, we tried to triangulate the interview data as extensively as possible among the mothers, providers, and staffs at health centers.”

3. Please include additional information regarding the interview guide used in the study and ensure that you have provided sufficient details that others could replicate the analyses. For instance, if you developed a questionnaire as part of this study and it is not under a copyright more restrictive than CC-BY, please include a copy, in both the original language and English, as Supporting Information. In addition, please provide any details of pre-testing.

Response) We have elaborated more on the interview method part including data, sample, and analysis as below (starting from line 126). We also have provided original and English versions of the semi-structured questionnaire we developed for the interview as supporting information.

“**Data** In-depth interviews were conducted in person by three authors alternately in pairs using a semi-structured questionnaire drawn up based on results from descriptive analyses and previous articles. Original questionnaires both in Korean and translated in English are included in a supplemental file to this manuscript (Supporting information file 1). Data were collected between November and December in 2016.

**Sample** Our interview sample composed of three groups: 1) mothers who did and did not use local maternal hospitals supported by the government were recruited using a purposive sampling strategy and interviewed to identify facilitators and barriers to the utilization of government-supported hospitals, named “demand-side factors”. Purposive sampling strategy is used when researchers assume that certain categories of individuals may have a unique, or important perspective on the phenomenon in question [19]. Since the authors did not have direct access to the patient information, we asked the government-supported hospitals to recruit mothers who recently had delivered a baby in their hospital. To recruit the mothers who had delivered outside their residential areas, we contacted and asked personnel in charge of maternal and child health in local health centers because they have almost every information on the pregnancies and births that had occurred within their territory. 2) Doctors, nurses and hospital managers in government-supported hospitals were interviewed to identify the difficulties in running the OB/GY services based on government funding, named “supply-side factors”. 3) Finally, personnel in charge of maternal and child health in local health centers in OUAs were additionally interviewed to examine the difficulties in implementing the government-support program. Doctors, nurses, and hospital managers and personnel in local health centers were recruited by convenience sampling. Convenience sampling might have a danger that generalization is unwarranted when the sample universe is broad. However, this concern would be insignificant because our interview covered 18 of 35 government-supported hospitals, which amount to more than 50% of the entire sample.

**Analysis** All the interviews were tape-recorded and transcribed. To maximize the rigor, three authors who conducted interviews independently read and coded the randomly allocated batches of interview transcripts using grounded theory methodology [20] and reached agreement on the emerging themes and subthemes through discussion. Specifically, the constant comparative method was employed to deduct barriers and promoters to the utilization of local government-supported maternal hospital in their residential area and difficulties in operating the support program. Each word and sentence were analyzed to identify tentative themes, which were compared with each other within and across transcripts. Themes with similar meanings were grouped together and finally, categories were formulated from the themes. We dropped the themes that were not within the scope of our immediate interest. “

 4. We note that you have indicated that data from this study are available upon request. PLOS only allows data to be available upon request if there are legal or ethical restrictions on sharing data publicly. For more information on unacceptable data access restrictions, please see <http://journals.plos.org/plosone/s/data-availability#loc-unacceptable-data-access-restrictions>.  In your revised cover letter, please address the following prompts:

a) If there are ethical or legal restrictions on sharing a de-identified data set, please explain them in detail (e.g., data contain potentially sensitive information, data are owned by a third-party organization, etc.) and who has imposed them (e.g., an ethics committee). Please also provide contact information for a data access committee, ethics committee, or other institutional body to which data requests may be sent.

Response) National Health Insurance claims big data that our analyses are based on is managed by National Health Insurance Service (NHIS). The database for the purpose of statistical analyses can be accessed by only authorized researchers through remote access system or big data center. The remote access system is to support for analysis through virtualized PC at the user’s desired location, and a total of 180 accounts are in operation. The Big data center is a space for data analysis for the research purpose where there are 40 seats in headquarters and branches of NHIS. Interest parties can contact NHIS to obtain the data in the following ways: Tel : 033) 736-2431, 2432, 2433 (information analysis department in big data operation room) Fax: fax033) 749-6337, Web : <https://nhiss.nhis.or.kr/bd/ab/bdaba013eng.do>

b) If there are no restrictions, please upload the minimal anonymized data set necessary to replicate your study findings as either Supporting Information files or to a stable, public repository and provide us with the relevant URLs, DOIs, or accession numbers. For a list of acceptable repositories, please see <http://journals.plos.org/plosone/s/data-availability#loc-recommended-repositories>.  We will update your Data Availability statement on your behalf to reflect the information you provide.

Response) The dataset we used for our study has a restriction. Please refer to the answer a)

5. Thank you for stating the following in the Competing Interests section:

"NO authors have competing interests"

We note that one or more of the authors are employed by a commercial company: Bronx Partners for Health Communities New York City

Response) Bronx Partners for Health communities (BPHC) is not a commercial company but is a non-profit organization. BPHC is a partnership of more than 200 health & community-based health service providers working to improve the health and well-being of Bronx residents by participating DSRIP (Delivery System Reform Incentive Payment) system. The BPHC has nothing to do with the whole process of this research including any form of funding (JRM did not receive any kind of compensation from BPHC with regard to author time for this research work), study design, or data collection, etc. The author affiliated with BPHC participated in this paper with pure academic interest and worked on this paper in her off-hour.

6.     Please provide an amended Funding Statement declaring this commercial affiliation, as well as a statement regarding the Role of Funders in your study. If the funding organization did not play a role in the study design, data collection and analysis, decision to publish, or preparation of the manuscript and only provided financial support in the form of authors' salaries and/or research materials, please review your statements relating to the author contributions, and ensure you have specifically and accurately indicated the role(s) that these authors had in your study. You can update author roles in the Author Contributions section of the online submission form.

Please also include the following statement within your amended Funding Statement.

“The funder provided support in the form of salaries for authors [insert relevant initials], but did not have any additional role in the study design, data collection and analysis, decision to publish, or preparation of the manuscript. The specific roles of these authors are articulated in the ‘author contributions’ section.” If your commercial affiliation did play a role in your study, please state and explain this role within your updated Funding Statement.

Response) The BPHC has nothing to do with this study throughout the whole process. JRM did not receive any kind of compensation from BPHC with regard to author time for this research work. She worked on this paper in her extra time. Therefore, the author’s role or contribution doesn’t need to be updated. (Please refer to the answer 5)

7. Please also provide an updated Competing Interests Statement declaring this commercial affiliation along with any other relevant declarations relating to employment, consultancy, patents, products in development, or marketed products, etc.

 Within your Competing Interests Statement, please confirm that this commercial affiliation does not alter your adherence to all PLOS ONE policies on sharing data and materials by including the following statement: "This does not alter our adherence to  PLOS ONE policies on sharing data and materials.” (as detailed online in our guide for authors <http://journals.plos.org/plosone/s/competing-interests>) . If this adherence statement is not accurate and there are restrictions on sharing of data and/or materials, please state these. Please note that we cannot proceed with consideration of your article until this information has been declared.

 Please include both an updated Funding Statement and Competing Interests Statement in your cover letter. We will change the online submission form on your behalf.

Response) We added the following: " The affiliation of BPHC does not alter our adherence to  PLOS ONE policies on sharing data and materials.”

8. Please know it is PLOS ONE policy for corresponding authors to declare, on behalf of all authors, all potential competing interests for the purposes of transparency. PLOS defines a competing interest as anything that interferes with, or could reasonably be perceived as interfering with, the full and objective presentation, peer review, editorial decision-making, or publication of research or non-research articles submitted to one of the journals. Competing interests can be financial or non-financial, professional, or personal. Competing interests can arise in relationship to an organization or another person. Please follow this link to our website for more details on competing interests: <http://journals.plos.org/plosone/s/competing-interests>

Response) Thanks for reminding. We confirm again that BPHC does not have any financial or non-financial, professional, or personal competing interest that might interferes with, or could reasonably be perceived as interfering with objective presentation, peer review, editorial decision-making or publication of the research article.

Reviewers' comments:

**Comments to the Author**
**Reviewer’s report:** Richard Kalisa MD, PhD

Thank you for the opportunity to review this manuscript

The manuscript provides an in-depth evaluation of the effectiveness of the government support program for obstetrically underserved areas in South Korea. It highlights the facilitators and barriers to use of government-supported hospital and the importance of the area-level in explaining the maternal health services utilisation in OUAs. The findings are useful to the program and could support its continued implementation and improvement. Overall, the paper is well-written. However, a few issues need consideration:

1. Qualitative arm of the study: The authors should provide details on the sampling of the study participants, rigour/trustworthiness of their qualitative study findings

Response) We appreciate for your careful comments on the qualitative study part. We have more elaborated on the composition of the sample and sampling method for each group of interview participants in subsection of “sample” in the “study 2: qualitative component” method part as below:

“**Sample** Our interview sample composed of three groups: 1) mothers who did and did not use local maternal hospitals supported by the government were recruited using a purposive sampling strategy and interviewed to identify facilitators and barriers to the utilization of government-supported hospitals, named “demand-side factors”. Purposive sampling strategy is used when researchers assume that certain categories of individuals may have a unique, or important perspective on the phenomenon in question [19]. Since the authors did not have direct access to the patient information, we asked the government-supported hospitals to recruit mothers who recently had delivered a baby in their hospital. To recruit the mothers who had delivered outside their residential areas, we contacted and asked personnel in charge of maternal and child health in local health centers because they have almost every information on the pregnancies and births that had occurred within their territory. 2) Doctors, nurses and hospital managers in government-supported hospitals were interviewed to identify the difficulties in running the OB/GY services based on government funding, named “supply-side factors”. 3) Finally, personnel in charge of maternal and child health in local health centers in OUAs were additionally interviewed to examine the difficulties in implementing the government-support program. Doctors, nurses, and hospital managers and personnel in local health centers were recruited by convenience sampling. Convenience sampling might have a danger that generalization is unwarranted when the sample universe is broad. However, this concern would be insignificant because our interview covered 18 of 35 government-supported hospitals, which amount to more than 50% of the entire sample.”

Also, we have added the process we adopted to ensure rigorousness and trustworthiness in subsection of “analysis” in the “study 2: qualitative component method” part as below:

**“Analysis** All the interviews were tape-recorded and transcribed. To maximize the rigor, three authors who conducted interviews independently read and coded the randomly allocated batches of interview transcripts using grounded theory methodology [20] and reached agreement on the emerging themes and subthemes through discussion. Specifically, the constant comparative method was employed to deduct barriers and promoters to the utilization of local government-supported maternal hospital in their residential area and difficulties in operating the support program. Each word and sentence were analysed to identify tentative themes, which were compared with each other within and across transcripts. Themes with similar meanings were grouped together and finally, categories were formulated from the themes. We dropped the themes that were not within the scope of our immediate interest.”

2. Provide illustrative quotes for supply-side barriers to the success of the program

Response) We did not include the quotes due to the excessive words count. We have now added the relevant quotes in the supply-side barrier section as below:

- From line 363 to 365 under the subtitle of “difficulty in recruiting health professionals”

*“We are having huge difficulties recruiting pediatrician, especially specialized in neonate. So we have to refer premature babies to bigger hospitals in the neighborhood district. However, even they don’t want to take premature baby patients (HC1KW).”*

- From line 373 to 375 under the subtitle of “insufficient fund”

*“Medical malpractice is more frequent in OB/GY department than other ones. Doctors have to bear the cost for their malpractice because we don’t have any insurance and the like. So we see patients in a very defensive way (DRKB2)”*

- From line 373 to 375 under the subtitle of “inadequacy of a performance indicator of the support program”

*“We think the value of this government-support program is that we can care for the mothers who cannot go far to hospitals due to various reasons no matter how small they may be in number. It is a government’s responsibility to make an environment where every pregnant woman can utilize the maternal service without geographical limitation. If the government stop the support just because it could not show a big increase in the number of delivery cases within the district, those women would lose their right for access to basic health service (MOHJN1)”*

3. In addition, under “preference for a specialised maternal hospital over a general hospital” they should label the quote appropriately.

Response) Now we have labelled it appropriately as below (line 333) :

*“XX hospital (government-supported maternal hospital) has a delivery room, newborn unit, and ICU located next to each other. In addition, pediatrics outpatient clinics are right next to the OB/GY inpatient wards, which can expose mothers who just delivered a baby to infection from outpatient* (*NOMOCB2)."*

4. In the discussion, it would be important to see a comparison between this study findings and previous evaluations conducted on the program. What are some of the similarities and differences among the different evaluations – findings and recommendations?

Response) Four rounds of evaluations were performed, of which two are mainly focused on designing maternal service delivery system for high-risk mothers and babies in OUAs rather than the evaluation of the government support program. Some findings identified in our study were also presented in previous evaluations, but with less emphasis. We have added the similarities and differences between previous evaluations and the current evaluation studies while describing the summary of findings of our study as below.

1) From line 457 to 462 as below:

“Although previous evaluations on government-support program have identified reliable quality of hospital facilities and system as one of the main factors for utilizing the hospital outside their district [12, 14], recommending to improve the quality of the government-supported hospital. none of the previous evaluations has not brought the changing preference of mothers for luxurious services or delivery culture among young mothers to their attention.”

2) From line 463 to 467 as below:

“Second, most of the interviewed mothers put much priority on the availability of post-partum care center nearly. Importance of post-partum care centers in mother’s choice of delivery hospital has grown, given the result of previous evaluation performed in 2014 that only small portion of mothers (less than 6%) cited a lack of post-partum care center as a reason of not using government-supported hospital.”

3) From line 528 to 533.

“First, we considered the compositional and contextual characteristics of area such as mother’s demographic, socioeconomic status in the area, fiscal self-reliance of the area, etc. when comparing the DCI between government-supported OUAs and comparison areas, while a series of previous evaluation performed only non-adjusted comparison of DCI ignoring the disadvantageous conditions of OUAs and presented a concern for still low DCI in OUAs.”

5. Results: Revise the sentence “Specifically….” to read better

Response) If the reviewer intended to point out the sentence starting with “specifically” in the phase 1: descriptive statistics (starting from line 174), we paraphrased the sentence to read more easily as below:

“Specifically, the number of deliveries that had happened in 71 districts belonging to the DCA category accounted for about 73.3% of the total delivery that happened across the country (393,826 deliveries in 266 districts in total), meaning that three-fourths of the deliveries happened in only one-fourth of the districts.”

6. Discussion: The recommendation on “…*just making their travel more comfortable by providing transportation itself or subsidizing transportation expenses rather than persuading them to use local hospital in their residential area would be more reasonable way to ensure accessibility while better serving their needs*.” might negate the importance of physical/geographical accessibility.

Response) We understand the reviewer’s concern. However, our suggestion for arranging transportation rather than inducing them to use the local hospital is only for mothers who have easy access to the hospital in a neighbouring city in terms of travel time. The local government-supported hospital might be in closer proximity but hospitals in a neighbouring city is still considered to be within the range of physical distance that mothers arrive safely enough even in the emergency. In this case, support for mother’s choice would be a way to respect their preference as well as ensure their safe delivery. In this sense, we would say that this suggestion does not negate the importance of physical /geographical accessibility. We paraphrased the relevant part to avoid any misunderstanding.

7. Also, could women still utilising city hospital be related to birth preparedness and complication readiness?

Response) The main reason why the mothers keep traveling to the neighbouring city is because they want to use the specialized maternal hospitals. Specialized maternal hospitals are accredited by the Ministry of Health to provide high-level medical service by appraising the quality of services based on several dimensions of indicators. Therefore, they are generally better-equipped and provide better service compared to others. Birth preparedness related to facility- and provider- level includes supplies, equipment, staffs, systems, or provider’s clinical competence.^[1]^ In this sense, city hospitals might even be better in birth preparedness and complication readiness.

8. The manuscript needs an entire revision of typographical errors

Response) We have had the native speaking researcher review and edit the whole manuscript after revision.

9. Add the study strength and suggestions for further studies

Response) We have added the strengths of our study as below (from line 527 to 536) :

“On the other hand, this study has several strengths that distinguish the present study from the previous evaluation and can provide policy implications. First, we considered the compositional and contextual characteristics of area such as mother’s demographic, socioeconomic status in the area, fiscal self-reliance of the area, etc when comparing the DCI between government-supported OUAs and comparison areas, while a series of previous evaluation performed only non-adjusted comparison of DCI ignoring the disadvantageous conditions of OUAs and presented a concern for still low DCI in OUAs. Adjusted comparison of DCI for a range of individual and area-level characteristics would enable achievement of a support program to be evaluated in a fair way. Another strength is that our analyses were based on not sample data but population data, which means there is no concern about generalizability.”

Also, suggestions for the further study was provided as below (from line 551 to 553):

“Further study is needed to explore the break-even point of the maternal hospitals where the hospital considers going out. This would provide information on the minimal amount of investment for government-supported hospital to meet the mothers’ demand.”

10. The authors should consider using PLoS ONE reference style

Response) Thanks. We have revised Reference part observing the PLoS One reference style.

1. JHPIEGO, Monitoring birth preparedness and complication readiness. Tools and indicators for maternal and newborn health. 2004. Baltimore, USA.
